# Left-Sided Partial Anomalous Pulmonary Venous Return Uncovered During Central Venous Catheterization in a Patient Undergoing Thoracic Surgery

**DOI:** 10.7759/cureus.89376

**Published:** 2025-08-04

**Authors:** Michail Thomaidis, Sofoklis Mitsos, Vasileios Leivaditis, Nikolaos Korodimos, Athanasios Papatriantafyllou, Efstratios N Koletsis, Periklis Tomos

**Affiliations:** 1 Department of Thoracic Surgery, Attikon General Hospital, National and Kapodistrian University of Athens, Athens, GRC; 2 Department of Cardiothoracic and Vascular Surgery, Westpfalz-Klinikum, Kaiserslautern, DEU; 3 Department of Cardiothoracic Surgery, General University Hospital of Patras, Patras, GRC

**Keywords:** central venous catheterization, incidental finding, interdisciplinary communication, left-sided pulmonary venous anomaly, partial anomalous pulmonary venous return (papvr), thoracic surgery, vascular anomaly

## Abstract

Left-sided partial anomalous pulmonary venous return (PAPVR) may remain clinically silent and undiagnosed until incidentally identified, potentially introducing complexity in perioperative assessment and management, particularly in patients with significant comorbidities. We report the case of a 77-year-old male with metastatic colorectal adenocarcinoma and a history of multiple right-sided pulmonary metastasectomies. He underwent a right completion upper bilobectomy. Postoperatively, placement of a central venous line in the left internal jugular vein (selected due to intraoperative accessibility, although the right side is generally preferred for central access) revealed unexpectedly high oxygen saturation levels, prompting an urgent computed tomography angiogram. Imaging confirmed a previously undiagnosed left-sided PAPVR, with drainage of the left upper pulmonary vein into the left jugular vein. The central line was removed and replaced on the contralateral side. No surgical correction was required due to the asymptomatic nature and contralateral location of the anomaly. The patient recovered uneventfully, aside from a brief episode of atrial fibrillation managed conservatively. This case highlights the importance of maintaining clinical awareness of vascular anomalies such as PAPVR in thoracic surgical patients. Incidental findings may have significant implications for central venous access, anesthetic management, and postoperative care. Thorough imaging review and effective interdisciplinary communication are essential to ensure optimal outcomes.

## Introduction

Partial anomalous pulmonary venous return (PAPVR), also known as partial anomalous pulmonary venous connection (PAPVC), is a rare congenital condition, accounting for less than 1% of all congenital heart disease [1]. It is characterized by the abnormal drainage of one or more pulmonary veins into the right atrium or the systemic venous circulation, rather than the left atrium. The estimated prevalence ranges between 0.4% and 0.7%, with left-sided PAPVR representing only about 10% of cases [2].

This condition may result from various congenital malformations, including anomalous connections between pulmonary veins and systemic veins such as the superior vena cava or jugular veins. The most common form of PAPVR involves a right upper lobe pulmonary vein that drains into the right atrium. In left-sided cases, the left upper pulmonary vein may drain into the left innominate vein, which ultimately empties into the right atrium. Other variants include drainage into the superior vena cava, coronary sinus, inferior vena cava, or azygos vein [3].

These abnormalities can lead to significant hemodynamic changes, including left-to-right shunting, elevated right atrial pressures, and complications such as pulmonary hypertension and right heart failure. PAPVR may occur in isolation but is frequently associated with other congenital cardiac anomalies, most notably sinus venosus atrial septal defect, which is present in a majority of cases. Other associations include cor triatriatum, reported in 10-33% of cases, and Turner syndrome, which has also been linked to an increased risk of PAPVR [4,5].

Many patients remain asymptomatic, especially when the shunting is minimal. However, as the condition becomes more pronounced, clinical manifestations may emerge during childhood or adulthood. These can include growth delays, exercise intolerance, recurrent respiratory infections, dyspnea, palpitations, tachycardia, heart murmurs, and chest pain. Diagnosis is often incidental but may also follow targeted investigation of such symptoms using imaging techniques like echocardiography, computed tomography (CT), or magnetic resonance imaging (MRI) [6,7].

Herein, we present a rare case of isolated left-sided PAPVR discovered incidentally in a postoperative oncology patient. This case highlights the diagnostic challenge such anomalies may pose and underscores the importance of interdisciplinary communication in perioperative care. We also briefly review the limited literature on this uncommon entity.

## Case presentation

A 77-year-old male with a history of metastatic colorectal adenocarcinoma was initially treated with a partial colectomy, followed by three pulmonary metastasectomies for right upper and middle lobe lesions at another institution. He had also undergone multiple cycles of chemotherapy and immunotherapy. During routine follow-up, a Positron Emission Tomography scan (PET scan) revealed hypermetabolic nodules in the remaining right upper and middle lobes. He was referred for a fourth thoracic procedure - a right completion upper and middle bilobectomy - which was performed uneventfully as planned.

**Figure 1:**
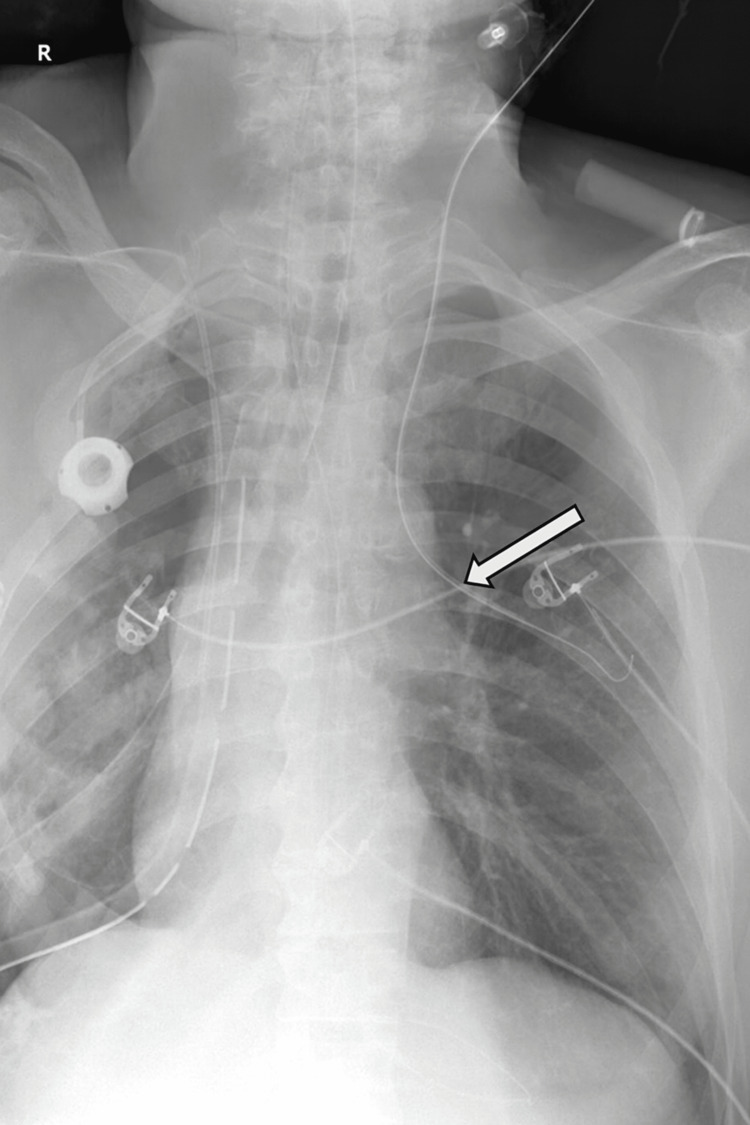
Postoperative chest X-ray demonstrating the central venous catheter positioned within an anomalous vascular structure (arrow), later identified as a left-sided partial anomalous pulmonary venous connection. The catheter tip appears unusually high and lateral, raising suspicion for malposition and prompting further investigation with computed tomography angiography.

**Figure 2:**
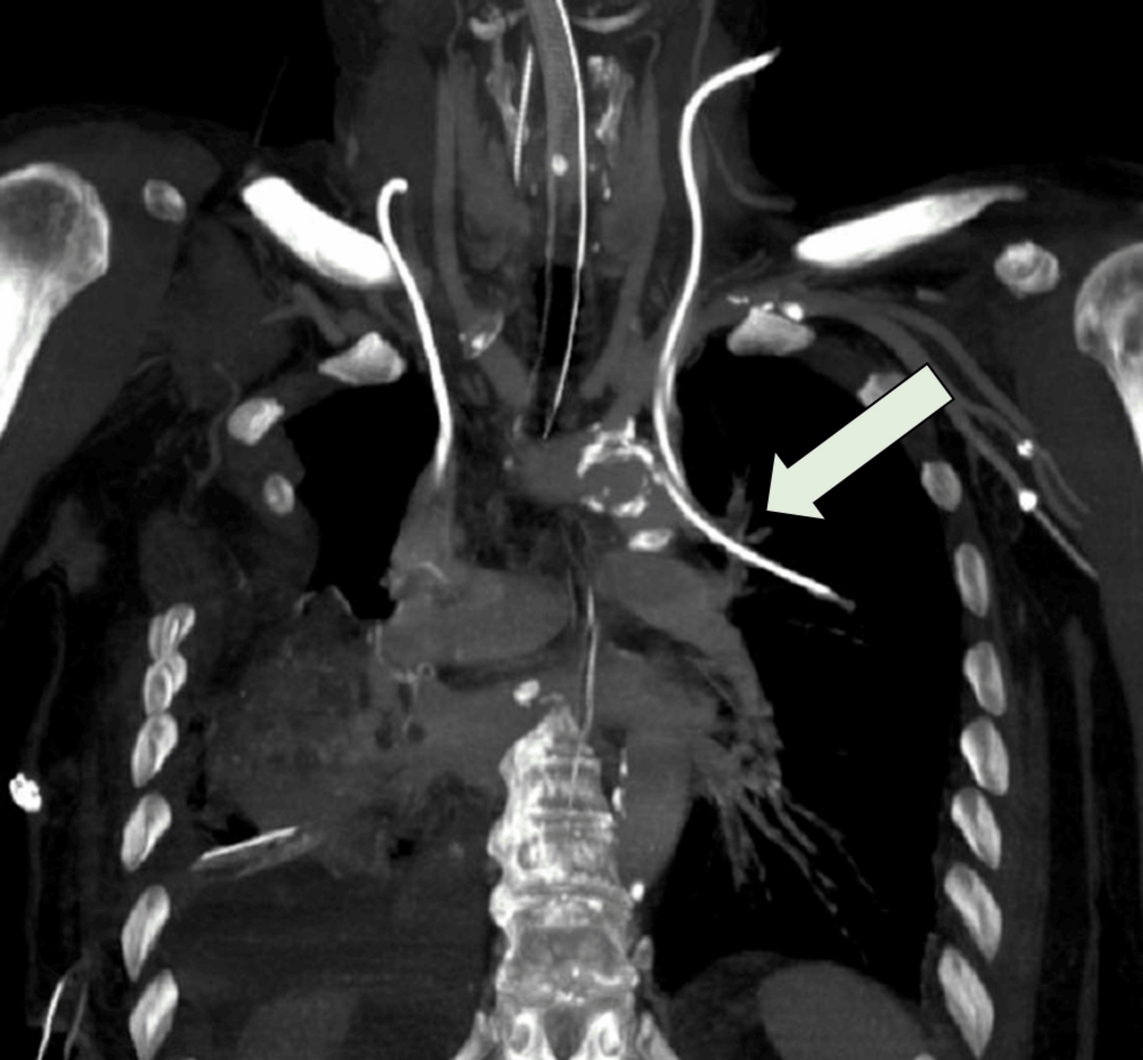
Coronal reconstruction from contrast-enhanced computed tomography angiography showing an anomalous vascular connection between the left upper pulmonary vein and the left internal jugular vein (arrow). The central venous catheter is visible within the anomalous vessel, highlighting the inadvertent placement in a pulmonary vein draining oxygenated blood into the systemic venous system.

**Figure 3:**
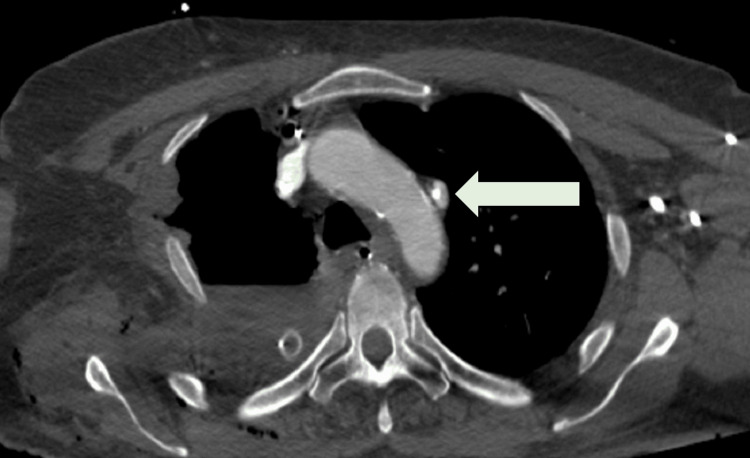
Axial (transverse) computed tomography angiogram slice demonstrating the anomalous venous return from the left upper pulmonary vein into the left internal jugular vein (arrow), with the central venous catheter seen within the aberrant vessel. This view confirms the unusual anatomy and explains the unexpectedly high oxygen saturation obtained from venous sampling.

Postoperatively, the patient was admitted to the intensive care unit (ICU) with two thoracic drainage tubes in place for standard monitoring. A central venous catheter was placed in the left internal jugular vein. Subsequent blood gas analysis from this line revealed an unexpectedly elevated partial pressure of oxygen (PaO₂) of 385 mmHg, a result inconsistent with typical systemic venous values. A postoperative chest X-ray revealed an unusual position of the central venous catheter, with its tip located high and lateral on the left side (Figure 1), raising suspicion for malposition. This prompted an urgent CT angiogram, which revealed an anomalous vascular connection between the left upper pulmonary vein and the left jugular vein (Figures 2, 3), consistent with a diagnosis of PAPVR. No other structural cardiac anomalies, such as atrial septal defect, were identified.

The central line was immediately removed and replaced via the right internal jugular vein, where no vascular anomalies were observed. The patient remained hemodynamically stable under ICU care. Given the asymptomatic nature of the PAPVR and its location contralateral to the operative field, no surgical intervention was deemed necessary.

He was subsequently transferred to the thoracic surgery ward. During recovery, he experienced a single episode of atrial fibrillation, which was successfully managed with antiarrhythmic medication (amiodarone 150 mg IV bolus over 10 minutes, then 1 mg/min for six hours, 0.5 mg/min for 18 hours; patient weight: 72 kg). Following the removal of thoracic drains and in the absence of further complications, the patient was discharged with outpatient follow-up.

## Discussion

Embryologically, PAPVR results from the failure of primitive pulmonary venous drainage to regress during fetal development [4]. Although PAPVR is a congenital defect, it often remains undiagnosed for years, particularly when asymptomatic. Many cases are discovered incidentally during imaging performed for unrelated conditions. Isolated left-sided PAPVR, as observed in our patient, is especially rare and often escapes detection unless imaging or interventional procedures bring it to light [7].

In clinical practice, left-sided PAPVR may be mistaken for other vascular anomalies unless actively considered in the differential diagnosis. One of the most important differentials is a persistent left superior vena cava (PLSVC), as both can appear as vertical anomalous veins lateral to the aortic arch on computed tomography [8]. Other conditions that may mimic PAPVR on imaging include an enlarged left superior intercostal vein, collateral venous pathways, scimitar syndrome, acquired systemic-to-pulmonary venous shunt, and pulmonary varix due to atresia or stenosis of a pulmonary vein leading to compensatory dilation of another [3].

Although such anomalies are typically diagnosed in childhood due to symptoms like exercise intolerance, recurrent respiratory infections, or heart murmurs, they may go undetected into adulthood, particularly in patients with complex oncological histories, as in our case. The presence of PAPVR can pose significant risks during thoracic surgical procedures, especially if the abnormal anatomy is not identified beforehand. Unrecognized anomalous veins may be inadvertently injured, potentially leading to hemorrhage or disrupted pulmonary circulation. This case underlines the diagnostic and management challenges posed by incidental findings of vascular anomalies. The anomalous venous return in our patient was revealed when a central venous catheter was placed in the left internal jugular vein, yielding abnormally high oxygen saturation levels inconsistent with venous blood. Emergency imaging subsequently identified the left-sided PAPVR. While no immediate harm resulted, this scenario highlights the potential for misleading clinical data and even harmful interventions if such anomalies go unrecognized. Central line placement through an anomalously draining vein may impair accurate venous blood sampling, drug administration, and hemodynamic monitoring [9]. This is particularly important when accessing the left internal jugular vein, where unrecognized venous anomalies such as PAPVR may increase the risk of catheter misplacement and compromise clinical decision-making [10].

Interestingly, a chest CT scan performed earlier in the patient's cancer workup had already demonstrated the anomalous vein, but this detail was not effectively communicated to the ICU team. This case therefore emphasizes the importance of thorough multidisciplinary communication and continuity of care, especially in patients with extensive medical records and complex clinical trajectories. Management of PAPVR must be tailored to each individual case. Surgical correction is typically reserved for symptomatic patients, those with significant left-to-right shunting, or those undergoing lung surgery on the same side as the anomalous connection. However, when PAPVR is present in a non-resected lobe, as highlighted by Matsui and Takami (2024), lobectomy may still increase the risk of postoperative right heart failure, and the potential need for surgical revascularization should be evaluated preoperatively [11]. Repair usually involves redirecting the anomalous pulmonary vein to the left atrium or addressing any associated structural defects, such as atrial septal defect. In contrast, asymptomatic patients with incidental findings-particularly when the anomaly is contralateral to the operative field-may be managed conservatively with observation. In our patient, surgical intervention was not indicated, as the PAPVR was located on the left, while the thoracic surgeries were performed exclusively on the right side [12,13].

Postoperatively, patients with known or corrected PAPVR should be monitored for potential complications, including cardiac arrhythmias, hemodynamic instability, and pulmonary issues. Altered pulmonary venous return can influence oxygenation and overall respiratory function. Our patient experienced a brief episode of atrial fibrillation during recovery, which was successfully managed with medication. Ultimately, this case reinforces several key points: the necessity of comprehensive imaging review before thoracic surgery, the value of interdisciplinary communication across surgical, radiologic, and ICU teams, and the importance of maintaining clinical suspicion for vascular anomalies that may influence perioperative management and patient safety.

## Conclusions

PAPVR is often an incidental finding but may have important implications for perioperative management. When identified pre- or intraoperatively, it can necessitate modifications to the surgical approach, anesthetic strategy, and postoperative monitoring. Surgeons, anesthesiologists, and ICU teams should remain vigilant for such vascular anomalies, particularly in patients undergoing unilateral thoracic procedures or those with complex medical histories. Comprehensive preoperative imaging review and effective interdepartmental communication are essential to ensure that anomalies like PAPVR are recognized and appropriately managed. Awareness of these rare anatomical variants allows for tailored surgical planning, reduces the risk of complications, and contributes to safer, more effective patient care.
